# Supplementary material for: Complexome profiling of the Chlamydomonas psb28 mutant reveals TEF5 as an early PSII assembly factor
Source: Plant Cell. 2025 Mar 17;37(6):koaf055. doi: 10.1093/plcell/koaf055 (PMC12164590; doi:10.1093/plcell/koaf055)
Supplement: koaf055_Supplementary_Data [file koaf055_supplementary_data.zip › psb28_interactive.zip!/plots/intensity/qualityPlots/peptides/TIC40_Cre12.g508000.t1.1.html]

### Cre12.g508000.t1.1

Trivial name: TIC40  
SeqCoverage [median]: 57.50  
Mass: 42.95 kDa  
  
 

| Sequence | MissedCleavage | Mass [Da] | wtIntensity | muIntensity | Score |
| --- | --- | --- | --- | --- | --- |
| AGGTSSSSGSGAAPQDTGVMTDMMESMLR | 0 | 2818.19 | 0.00 | 3472.05 | 82.09 |
| EPSSSGSSSGASSSGR | 0 | 1425.60 | 414.16 | 651.50 | 108.59 |
| GMDFNQDKVNK | 1 | 1294.60 | 636.49 | 5038.52 | 89.81 |
| KPEPAPAAAAAGAAAAASSATKPLDVVEPVVK | 2 | 2954.61 | 2648.11 | 17267.40 | 110.91 |
| KPEPAPAAAAAGAAAAASSATKPLDVVEPVVKEPSSSGSSSGASSSGR | 3 | 4362.19 | 4036.89 | 41966.58 | 120.13 |
| LNSEGREPR | 1 | 1056.53 | 10479.71 | 23523.07 | 137.95 |
| MLYPYLPEPMR | 0 | 1408.69 | 0.00 | 4060.02 | 86.01 |
| QFAELGLKPEDVISK | 1 | 1672.90 | 6151.15 | 19917.43 | 59.71 |
| QQYYMAAPAAAYYPPPPAPYK | 0 | 2357.12 | 0.00 | 4078.48 | 80.86 |
| SSFFADVDAEASSR | 0 | 1487.65 | 3069.29 | 9486.97 | 138.94 |
| TPQQIMMEMAMK | 0 | 1437.65 | 5184.63 | 14046.79 | 130.10 |
| VLEKVTEIFQPQMGGQK | 1 | 1931.02 | 748.70 | 0.00 | 51.15 |
| VMANPDLAAGFSNPK | 0 | 1530.75 | 8355.48 | 27933.43 | 201.48 |
| VQAAIMDISQNPMNIVK | 0 | 1870.96 | 1560.15 | 30504.12 | 131.72 |
| VQEYMKNPK | 1 | 1135.57 | 4183.95 | 9652.34 | 72.64 |
| VTEIFQPQMGGQK | 0 | 1461.73 | 3417.76 | 10396.27 | 208.33 |
| YQTDPEIMK | 0 | 1123.52 | 3442.53 | 5272.47 | 156.35 |
| YQTDPEIMKVLEK | 1 | 1592.81 | 0.00 | 1159.85 | 54.20 |
|  |  |  | 54329.0 | 228427.3 |  |
